# Can platform use patterns be an indicator of HIV-related risk and sub-group heterogeneity among men who have sex with men in Singapore: a latent class analysis

**DOI:** 10.3389/fpubh.2024.1330282

**Published:** 2024-04-26

**Authors:** Isabel Tavitian-Exley, Ying Hao, Mark I-C. Chen, Chen Seong Wong, Chronos Kwok, Matthias Paul Han Sim Toh

**Affiliations:** ^1^National Public Health and Epidemiology Unit, National Centre for Infectious Diseases, Singapore, Singapore; ^2^National HIV Programme, National Centre for Infectious Diseases, Singapore, Singapore; ^3^Action for AIDS Singapore, Singapore; ^4^Saw Swee Hock School of Public Health, National University of Singapore, Singapore

**Keywords:** human immune deficiency virus, sexualised substance use, men who have sex with men, latent class modelling, sexual risk, risk behaviours, meeting platforms

## Abstract

**Introduction:**

Low-level HIV epidemic settings like Singapore face the challenge of reaching men at-risk who have less contact with programmes. We investigated patterns of meeting platform use by men seeking male sexual partners (MSM) as potential marker of risk to differentiate sub-groups for interventions.

**Methods:**

Latent Class Analysis (LCA) was applied to a survey sample of MSM recruited from bars/clubs, saunas and a smartphone application, using purposive sampling. The best-fit LCA model which identified homogeneous sub-groups with similar patterns of meeting platform was factored in multivariable regression to identify associations with risk behaviors on the pathway to HIV infection.

**Results:**

Overall 1,141 MSM were recruited from bars/clubs (*n* = 426), saunas (*n* = 531), and online (*n* = 184). Five patterns emerged, reflecting salient platform use characteristics: Sauna-centric (SC; *n* = 413), App-centric (AC; *n* = 276), Multiple-platforms (MP; *n* = 123), Platform-inactive (PI; *n* = 257), and “Do not hook up” (DNH; *n* = 72) classes. Men in the SC and MP classes had high probabilities of using saunas to meet partners; SC were older and less likely to have disclosed their sexual orientation. The MP class had high probabilities of connecting across all platforms in addition to saunas and more likely to have disclosed their sexual orientation, than the PI class. Men in the SC and MP classes had twice the odds of reporting multiple sex partners (aOR^SC^ = 2.1; 95%CI: 1.33.2; aOR^MP^ = 2.2; 95%CI: 1.14.6). Single/non-partnered MSM and those using alcohol/drugs during sex had 1.7 (95%CI: 1.22.5) and 3.2 (95%CI: 2.05.1) the odds respectively, of reporting multiple sex partners. The SC and MP classes had higher odds of engaging in group sex while MSM using alcohol/drugs during sex had twice the odds of reporting group sex. Alcohol/drugs and group sex were independently associated with condomless sex (as was lower education). Group sex, alcohol/drugs during sex, disclosure of sexual orientation or being Singaporean/permanent resident were associated with recent testing for HIV.

**Discussion:**

The five distinct risk profiles identified can help tailor differentiated HIV interventions—combined with field knowledge and other prevention—to expand HIV self-testing, Pre-Exposure Prophylaxis and other services (e.g., Mpox vaccination) to sub-groups at risk.

## Introduction

In Singapore, nine out of 10 newly reported HIV infections are diagnosed in men, half of which are attributed to homosexual transmission (1). Despite representing an estimated 2–10% of the male population in Singapore, men who have sex with men (MSM) have consistently accounted for 50% of newly reported HIV infections since 2015 and are a major and disproportionate contributor to the HIV epidemic (1–3).

In the context of competing public health concerns, ending the AIDS epidemic requires a robust understanding of socio-behavioural determinants of infection to better reach sub-groups disproportionately exposed to HIV and other sexually transmitted infections (STIs) with appropriate prevention messaging, interventions, and treatment (4–8).

Behaviours that are stigmatised in society may lead individuals to conceal certain practises and orientations. MSM, in particular, had limited opportunities to meet other recognisable MSM and sought to meet others on platforms or venues that were discreet or friendly (9). In the past two decades, ways for men to meet other men have increased from physical venues like sex-on-premise establishments [“saunas,” bars/clubs, public spaces, and private parties to online platforms, including internet chatrooms and dedicated smartphone applications (9, 10)]. The explosion of online platforms, many of which cater to specific sub-communities, has added complexity to sexual networks and amplified the challenges for prevention as it makes reaching population groups with appropriate and timely prevention messaging and treatment difficult.

Studies examining associations between meeting or interview locations and HIV infection risk have found different but inconsistent demographic profiles and/or risk behaviours between traditional and internet-based meeting platforms—where platforms may include physical (i.e., saunas, bars/clubs) or virtual spaces such as smartphone applications and chatrooms (9, 11). However, no published studies have examined *patterns* across all platforms used and none to date among MSM in Singapore.

We investigated the patterns of use of meeting platforms on which MSM find their sexual partners as potential marker(s) of HIV risk to differentiate sub-groups among them. By understanding distinct patterns of platform use, their determinants and associations may therefore provide public health programmes with added granularity to target interventions and services to specific sub-groups at risk of HIV.

The aims of our study were (i) to empirically identify meeting platform use classes based on the combination of the platform type reported and (ii) to investigate whether sexual and other risk behaviours, such as demographic factors and HIV infection, are associated with certain classes of meeting platforms among MSM.

## Methods

### Participants and recruitment

The seroprevalence survey among MSM has been conducted since 2007 as part of HIV surveillance in Singapore. The ninth survey round was conducted from December 2020 to July 2021 by the community-based organisation Action for AIDS (AfA), in partnership with the Department of Sexually Transmitted Infections Control Clinic (DSC) and National Centre for Infectious Diseases. Purposive sampling was used to recruit 1,300 MSM identifying as gay, bisexual, or of queer sexual orientation, aged 18 years and above, from physical venues frequented by MSM including five bars/clubs, five saunas, and the smartphone dating app Jack'd. The sample size was determined by previous seroprevalence and programme budget (Supplementary Table 1).

This round offered HIV and syphilis testing to address the growing epidemic of infectious syphilis among MSM (12). All eligible participants were offered on-site testing for HIV and syphilis after completion of informed consent and a structured self-administered questionnaire. Single-use finger prick test kits were used to collect blood samples, such as Gen 4 Rapid test kits from Alere Determine™ for HIV infection and rapid tests by Alere Determine™ for syphilis. The survey included basic socio-demographic questions on age, educational attainment, marital and residency status, employment, ethnicity, and nationality as well as sexual behaviours and use of programme services. The structured confidential questionnaire was completed on a tablet in physical locations and online for those registering through the smartphone app. Help from trained fieldworkers was available for those who required clarifications or assistance with language or terminology.

### Measures

Variables examined included social and demographic variables, disclosure of sexual orientation, and behavioural and serological markers of HIV and syphilis infection. Selected risk behaviours associated with HIV transmission included multiple sex partners in last 6 months, group sex in the last 6 months, substance use during sex, and condom use for anal sex in the last 6 months. Protective behaviours included recent testing for HIV (last 12 months) and knowledge of HIV pre-exposure prophylaxis (PrEP) (13–16).

Nine binary observed variables indicating different platforms used by MSM to meet other male sex partner(s) from the survey question “Where/how do you typically meet male sex partner” were used in the latent class analysis (Supplementary Table 2). The platform options included bars and clubs, saunas, public spaces (e.g., parks, gyms, or restrooms), private or home parties, internet sites and chatrooms, smartphone apps, or friends or paying someone to have sex (e.g., escorts and masseurs).

### Statistical analyses

We conducted a secondary analysis of data of a recent seroprevalence survey taken among MSM in Singapore using a combination of methods. Latent class analysis (LCA) is a form of latent class modelling (LCM) used to identify sub-groups of men with similar patterns in the platforms used by them to meet male sex partners. Originally developed for use in psychology and sociology research, latent class modelling methods were refined in the 1990's to explain respondent heterogeneity in survey response patterns involving dichotomous items (17, 18). LCA has increasingly been used in implementation research to empirically identify classes of individuals, based on a set of observed characteristics (18–22).

The LCA methodology can identify underlying relationships in a defined set of observed variables by grouping observations that display similar response patterns on observed variables and thus divide a heterogeneous population into more homogenous subgroups (i.e., latent classes) (19, 20). This method was selected above others such as cluster analysis or factor analysis for its ability to generate model-based class characterisations with conditional probabilities.

The selection of the best latent class model was informed by several fit statistics, epidemiology, meaningfulness, and practical implications of classes. The fit statistics examined were Pearson's chi-squared, log likelihood ratio tests (LR), and the Akaike (AIC) and Bayesian information criteria (BIC).

We compared socio-demographic, programme, and HIV risk behaviour variables across emergent classes using the best fit latent class model identified using univariable multinomial logistic regression (23, 24). Pearson's chi-squared test for categorical variables and Wald test *p*-values for coefficients in multinomial regression (i.e., log odds of each latent class) were derived.

Key behavioural outcomes on the pathway to HIV infection were examined in logistic regression as the low number of positive cases did not allow sub-group analysis by serological status. The outcomes of interest were as follows: multiple sex partners in the last 6 months, group sex in the last 6 months, unprotected anal sex in the last 6 months, and HIV testing in the last 12 months. We used multivariable logistic regression with robust sandwich variances to derive adjusted odds ratios (25). Multivariable regression models were adjusted for age, ethnicity, education, marital status, nationality, employment, recent HIV testing, and substance use during sex based on *a priori* knowledge and HIV transmission frameworks (26).

The predicted probabilities of reporting each behavioural outcome were then plotted by age as a continuous variable using the logistic regression model results for four behavioural outcomes: multiple sex partners, group sex, unprotected sex, and recent HIV testing. No outcome data were missing, and a complete case analysis was performed. The results are presented following guidelines outlined in the strengthening the reporting of observational studies in epidemiology (STROBE) (27, 28). Statistical analyses were performed using the R Statistical Software (v4.1.2; R Core Team 2021) and STATA Statistical Software version 16 (29–31).

### Ethical approval and consent to participate

Consent was sought at inclusion from eligible participants via a survey form and information sheet. The survey is anonymous, does not collect identifiable data, and is conducted as part of national HIV surveillance under the Infectious Diseases Act (32).

## Results

A total of 1,141 MSM aged 18–72 years (mean 33.8, SD 10.8) were recruited from bars/clubs (*n* = 426), saunas (*n* = 531), and smartphone dating applications (*n* = 184). Overall sample characteristics are summarised in Supplementary Table 3. Most respondents were single, separated, or divorced (81%), and 82% of them were in full- or part-time employment. Most were Singaporean (68%), a quarter had completed A levels or polytechnic education (24%), and over half completed university education (61%). Almost 20% had not disclosed their sexual orientation, and 38% had disclosed it to one group only. Multiple sex partners were reported by 79% of respondents, group sex by 34%, and alcohol and drug use during sex by 26%. Over half of the respondents reported unprotected anal sex (51%), 0.7% tested positive for HIV, and 1.1% were reactive for syphilis. Most participants (94%) reported using at least one of the nine platforms to meet male sex partners.

### Latent class model and meeting platform class membership

We fitted latent class models to the data and compared different fit statistics for two-, three-, four-, five-, and six-class models. Model fit indices including Pearson's chi-squared, Akaike information criterion (AIC), Bayesian information criterion (BIC), and likelihood ratio tests suggested a better fit for the five-class model (Supplementary Table 4), while a review of entropy and interpretability confirmed the five-class model solution shown in Table 1.

**Table 1:** Conditional probabilities^a^ of meeting platform use by class membership (five-class model).

**Latent class model**	**Class 1: Sauna-centric (** ***n*** **, %)**		**Class 2: App-centric (** ***n*** **, %)**		**Class 3: Multiple platforms (** ***n*** **, %)**		**Class 4: Platform-inactive (** ***n*** **, %)**		**Class 5: Do not hook-up (** ***n*** **, %)**		**All classes (** ***n*** **, %)**	
**Class probabilities**	413	100%	276	24%	123	11%	257	23%	72	7%	1,141	100%
**Meeting platform reported** ^b^												
Sauna	413	36%	91	33%	122	99%	38	15%	0	0%	573	50%
App	142	34%	276	100%	110	89%	91	35%	0	0%	619	54%
Internet	38	9%	0	0%	78	63%	91	35%	0	0%	207	18%
Bars and clubs	53	13%	0	0%	63	51%	56	22%	0	0%	172	15%
Friends	0	0%	53	19%	62	50%	122	48%	0	0%	237	21%
Parties	22	5%	0	0%	71	58%	59	23%	0	0%	152	13%
Public places	40	10%	10	4%	79	64%	10	8%	0	0%	149	13%
Paid for sex (e.g., escort)	8	2%	6	2%	29	24%	5	2%	0	0%	48	4%
Did not hook up (no platform used)	0	0%	0	0%	0	0%	0	0%	72	100%	72	6%

a Conditional probabilities are the probability that an MSM is in a given class, conditional on their answer “yes” to a specific platform use question (i.e., observed variable). Conditional probabilities are plotted in Figure 1 for each class of the five-class model.

b Column totals.

The largest class (class 1) in absolute numbers included 36% (*n* = 413) of the MSM sample followed by classes 2 and 4, which represented 24% (*n* = 276) and 23% (*n* = 257) of the sample, while the two remaining classes contributed 11% (class 3, *n* = 123) and 6.5% (class 4, *n* = 72), respectively. The rows in Table 1 show the conditional probabilities (in the five-class model) of endorsing a platform use variable for an individual classified in their most likely class. Most class-specific response probabilities for the (binary) observed variables were above 0.70 or below 0.30, suggesting similar item responses for participants in the same class and thus within-class homogeneity (33). Characteristics of meeting platform use and combinations for each class in the five-class model are illustrated in Figure 1.

**Figure 1:**
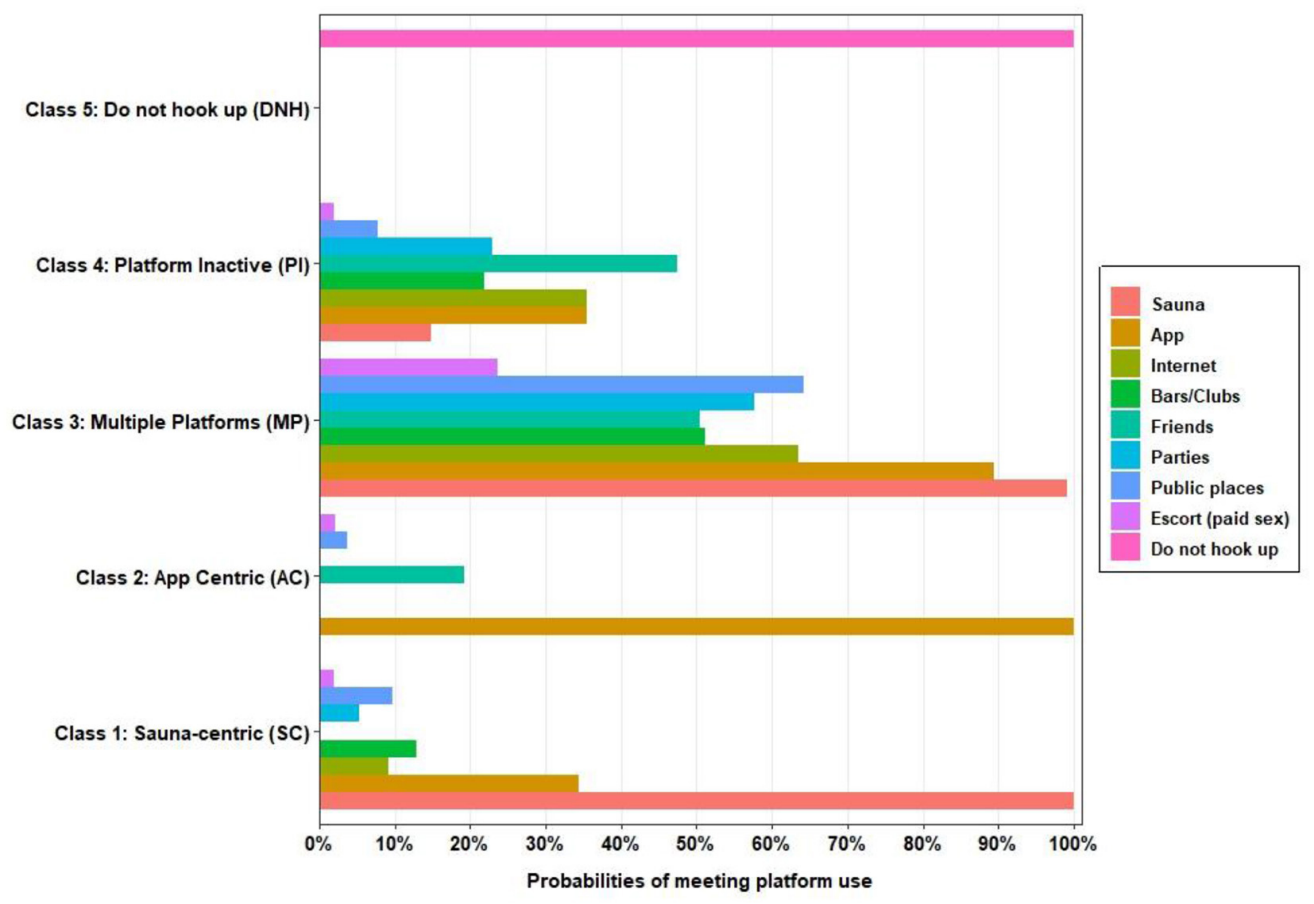
Platform use profiles among men who have sex with men for five-class model solution. The estimated probabilities for meeting platform use are plotted based on latent class (C1–C5) membership in Table 1. The horizontal axis (0–100%) shows the probability of meeting platform use for each platform. For example, the sauna-centric class (C1) had a 100% probability of using the sauna as a main platform and very low probabilities of using other platforms to meet other men. However, men in the “multiple platforms” class also had high probabilities of meeting other men in saunas and also high probabilities of connecting via the internet or smartphone applications as well as using all other meeting platforms. The nine observed variables are shown for clarity.

The five-class model used in subsequent multivariable analysis suggests five distinct patterns of use of meeting platforms with salient class features among them (Figure 1). Participants in class 1 (*n* = 413) had 100% probability of using the sauna as the preferred meeting platform, 34% probability of using a dating app, and low probabilities of using other meeting platforms such as bars and clubs (13%), internet (9%), and public places (9.8%). This class is referred to as the *sauna-centric (SC) class*. All participants in class 2 (*n* = 276) reported using a smartphone application as a platform to connect for sex, although 19% also reported meeting through friends and 4% in public places. This class is referred to as the *app-centric (AC) class*. Class 3 (*n* = 123) reported high probabilities (≥50%) of meeting on all meeting platforms and is subsequently referred to as the *multiple-platforms class (MP)*. Class 4 (*n* = 257) reported mostly low probabilities (≤35%) of using diverse meeting platforms and is referred to as the *Platform-inactive class (PI)*. Participants in Class 5 did not report using any platform to meet other men for sex and are referred to as the “*Do not hook up” (DNH)* class.

### Correlates of class membership

Univariable comparisons of socio-demographic characteristics, sexual risk behaviours, protective behaviours, and HIV prevalence between meeting platform classes were examined and are shown in Table 2. There were noteworthy differences between classes and the sample average in terms of education, marital status, employment, and sexual disclosure but not in terms of ethnicity or nationality.

**Table 2:** Univariable comparisons of socio-demographics, service characteristics, and HIV risk behaviours across latent classes.

**Variables (col %**, ***n*****)**		**All MSM (*n* = 1,141)**	**Class 1 Sauna-centric (*n* = 413)**	**Class 2 App-centric (*n* = 276)**	**Class 3 Multiple Platforms (*n* = 123)**	**Class 4 Platform inactive (*n* = 257)**	**Class 5 Do not hook up/”faithful” (*n* = 72)**	***X*^2^ *p*-value**
Age (mean, min-max)		33 (18–72)	38 (18–70)	30 (18–61)	32.5 (20–55)	32.3 (18–71)	33 (18–67)	<0.001 (ANOVA)
Age (years)	16–25	21% (234)	11% (46)	28% (77)	25% (31)	22% (57)	32% (23)	<0.001
	26–30	25% (290)	22% (89)	34% (94)	17% (21)	27% (69)	24% (17)	
	31–35	21% (238)	20% (83)^*^	21% (57)	24% (29)	22% (56)	18% (13)	
	36–40	12% (141)	13% (52)	9% (25)^*^	19% (23)	14% (37)	6% (4)^*^	
	41–50	13% (151)	19% (80)^*^	8% (21)	13% (16)	11% (27)	10% (7)	
	50+ years old	8% (87)	15% (63)^*^	1% (2)^*^	2% (3)	4% (11)	11% (8)	
Education	PSLE/O-Levels/ITE	15% (170)	24% (86)	7% (19)^*^	10% (12)	17% (42)	17% (11)	<0.001
	A Levels/polytechnic, and diploma	24% (261)	24% (94)	25% (68)	23% (27)	22% (54)	27% (18)	
	University and above	61% (673)	55% (220)	68% (186)	67% (80)	61% (150)	56% (37)	
Nationality	Singaporean	68% (771)	66% (271)	71% (195)	72% (89)	65% (167)	68% (49)	0.921
	Singapore PR	14% (160)	14% (56)	13% (37)	13% (16)	16% (40)	15% (11)	
	Malaysian	9% (106)	10% (43)	7% (20)	7% (9)	11% (28)	8% (6)	
	Filipino	4% (42)	5% (20)	3% (9)	2% (3)	4% (9)	1% (1)	
	Others	5% (62)	6% (23)	5% (15)	5% (6)	5% (13)	7% (5)	
Marital status	Single/separated/divorced	81% (919)	85% (351)	79% (218)	79% (97)	80% (205)	67% (48)	0.005
	Partnered/married	20% (222)	15% (62)	21% (58)	21% (26)	20% (52)	33% (24)^*^	
Employment	Full/part-time employment	81% (898)	86% (346)	75% (200)	78% (93)	85% (210)	74% (49)	<0.001
	Student	12% (136)	6% (24)^*^	21% (55)^*^	14% (17)	12% (29)	17% (11)	
	Unemployed/retired	0.2% (68)	8% (32)^*^	5% (12)	8% (9)	4% (9)	9% (6)	
Disclosure	No disclosure	19% (219)	27% (113)^*^	11% (30)^*^	11% (14)	14% (37)	35% (25)^*^	<0.001
	One group	39% (442)	43% (179)	33% (90)^*^	30% (37)^*^	44% (113)	32% (23)	
	≥ Two groups	42% (480)	29% (121)	57% (156)	59% (72)	42% (107)	33% (24)	
Risk behaviours	Multiple (≥2) sex partners	79% (904)	85% (352)^*^	79% (219)	91% (112)^*^	77% (197)	33% (24)^*^	<0.001
	Unprotected anal sex	51% (581)	49% (203)	50% (138)	57% (70)	52% (134)	50% (36)	0.635
	Engaged in group sex	34% (392)	39% (162)	26% (71)	50% (62)^*^	33% (85)	17% (12)^*^	<0.001
Substance use during sex	Alcohol and drugs	26% (298)	25% (103)	28% (78)	46% (57)^*^	21% (54)	8% (6)^*^	<0.001
	Alcohol only	8% (93)	4% (17)^*^	8% (21)	11% (14)	14% (35)	8% (6)	
	None	66% (750)	71% (293)	64% (177)	42% (52)	65% (168)	83% (60)	
Testing and treatment	Test for HIV in past 12 months	61% (692)	58% (238)	65% (180)	62% (76)	62% (159)	54% (39)	0.241
	Test for syphilis in last 6 months	30% (339)	29% (119)	31% (85)	33% (40)	31% (80)	15 (21%)	0.442
	STI diagnosis in past 6 months	9% (105)	10% (42)	8% (23)	11% (14)	9% (24)	3% (2)	0.290
Pre-exposure Prophylaxis (PrEP)	Has heard of PrEP	90% (1,022)	84% (346)^*^	96% (264)	94% (115)	93% (240)	79% (57)^*^	<0.001
	Has taken PrEP	22% (247)	23% (93)	18% (49)	24% (32)	24% (61)	17% (12)	0.219
Serological markers	HIV test positive	0.7% (8)	1% (4)	0.4% (1)	0.8% (1)	0.4% (1)	1.4% (1)	0.782
	syphilis reactive	1.1% (12)	0.7% (3)	0.4% (1)	1.6% (2)	1.2% (3)	4.2% (3)	0.066
	HIV +ve or syphilis reactive	1.7% (19)	1.5% (6)	0.7% (2)	2.4% (3)	1.6% (4)	5.6% (4)	0.068

Multinomial regression model. Missing values on variables Education (*n* = 37), Employment (*n* = 39). HIV, human immune-deficiency virus; STI, sexually transmitted infection; PrEP, pre-exposure prophylaxis; PSLE, primary school leaving examination (equivalent to primary school education); ITE, Institute of Technical Education (equivalent to secondary school education), PR, permanent resident.

The values with an “*” indicate variables for which significance was *p* < 0.05.

Men in the *SC class* (1) tended to be older, with a mean age of 38 years (min–max: 18–72), had lower educational levels (24% PSLE/O-levels/ITE vs. 15%), lower rates of disclosure (29 vs. 42%), and lower knowledge of PrEP (84 vs. 90%). Men in the *AC class* (2) primarily met sex partners online, were younger, with a mean age of 30 years, and were more likely to have completed university education (68 vs. 61%).

Participants in the *multiple platform* class (3) had a mean age of 32 years (min–max: 20–55), and a higher percentage of these participants had completed university education (68 vs. 61%), disclosed their sexual orientation to two groups or more (59 vs. 42%), reported multiple sex partners (91 vs. 79%), engaged in group sex (50 vs. 34%), and used substances during sex (47 vs. 26%). The *platform-inactive class* (4) did not differ significantly in terms of age, marital status, employment, or knowledge of PrEP. More MSM in Class 5 *(do not hook up)* were in a partnership or married (33 vs. 20%), were students (17 vs. 12%), unemployed (9 vs. 0.2%), and had not disclosed their sexual orientation (35 vs. 19%).

After adjusting for age, ethnicity, education, nationality, employment, marital status, sexual orientation disclosure, recent voluntary HIV testing, and substance use during sex, several factors were independently associated with each of the four behavioural risk outcomes. The results are shown in Tables 3–6.

**Table 3:** Model 1 outcome: multiple sex partners in last 6 months.

		**aOR**	**95% CI**	***p*-value**
Latent class (*ref. Platform inactive)*	Sauna-centric	2.08	(1.34–3.22)	0.001^*^
	App-centric	1.11	(0.71, 1.75)	0.640
	Multiple platforms	2.20	(1.07, 4.59)	0.030^*^
	Do not “hook up”	0.18	(0.09, 0.35)	<0.001^*^
Age (years) *(ref. 16–25 years)*	26–30	0.78	(0.42, 1.48)	0.450
	31–35	1.12	(0.58–2.16)	0.750
	36–40	0.66	(0.32–1.38)	0.270
	41–50	0.85	(0.41–1.75)	0.660
	50+	0.38	(0.17–0.87)	0.020^*^
Ethnicity *(ref. Chinese)*	Malay	1.59	(0.71, 3.54)	0.260
	Indian	1.38	(0.56, 3.41)	0.490
	Others	1.14	(0.51, 2.56)	0.761
Nationality *(ref. Singaporean)*	Singapore PR	1.62	(0.93, 2.80)	0.093
	Malaysian	0.85	(0.47, 1.54)	0.590
	Filipino	0.51	(0.19, 1.38)	0.190
	Others	0.63	(0.30, 1.32)	0.220
Education completed (*ref. University*)	A levels/polytechnic diploma	0.83	(0.53, 1.29)	0.399
	PSLE O levels/ITE diploma	0.60	(0.37, 0.97)	0.040^*^
Employment *(ref. employed full/part-time)*	Student	1.14	(0.58, 2.26)	0.710
	Unemployed/retired	1.82	(0.81, 4.08)	0.150
Marital status *(ref. married/partner)*	Single/separated/divorced/widower	1.70	(1.16, 2.50)	0.010^*^
Known HIV status *(ref. No)*	Yes	1.26	(0.87, 1.81)	0.220
Substance used during sex (*ref. none)*	Alcohol and drugs	3.24	(2.04, 5.13)	<0.001^*^
	Alcohol only	1.23	(0.68, 2.22)	0.498

Logistic regression model factoring in latent class was adjusted for age, ethnicity, education, nationality, employment, marital status, recent HIV testing, and substance use during sex (37 observations were dropped due to missing data). aOR, adjusted odds ratio; PR, permanent resident; PSLE, primary school leaving examination; O-level, ordinary Level; ITE, Institute of Technical Education. The values with an “*” indicate variables for which significance was *p* < 0.05.

Compared to the *PI* class, the odds of reporting multiple sex partners were highest in the *SC* (aOR = 2.1; 95% CI: 1.3–3.2) and *MP* classes (aOR = 2.2; 95% CI: 1.1–4.6; Table 3). Factors that were positively associated with multiple sex partners included being single/non-partnered (compared to married/partnered) and using alcohol and drugs during sex (compared to using none) with an odds ratio of 1.7 (95% CI: 1.2–2.5) and 3.2 (95% CI: 2.0–5.1), respectively. Being aged 50 years and above (compared to 25 and below) and completing PSLE, O-levels, or ITE diploma (compared to university-level education) were negatively associated with multiple sex partners (aOR = 0.6; 95% CI: 0.4–0.9).

SC or MP class members had greater probabilities of engaging in group sex in the past 6 months (aOR = 1.6; 95% CI: 1.1–2.3 and aOR = 1.7; 95% CI: 1.1–2.8, respectively), while AC and DNH classes had lower odds of engaging in group sex (aOR = 0.6; 95% CI:0.4–0.9 and aOR = 0.4; 95% CI: 0.2–0.8, respectively) (Table 4). Participants using alcohol and drugs or using alcohol-only during sex had twice the odds of reporting group sex (aOR = 2.2; 95% CI: 1.6–3.0 and aOR = 1.8; 95% CI: 1.1–2.8, respectively) compared to those using no substances. The model suggested that being aged 50 years or more and being of Malay ethnicity were negatively associated with group sex (aOR = 0.5; 95% CI: 0.3–1.0).

**Table 4:** Model 2 outcome: engaged in group sex in last 6 months.

		**aOR**	**95% CI**	***p*-value**
Latent class (*ref. Platform inactive)*	Sauna-centric	1.59	(1.11, 2.27)	0.010^*^
	App centric	0.59	(0.39, 0.88)	0.010^*^
	Multiple platforms	1.73	(1.06, 2.83)	0.030^*^
	Do not “hook up”	0.36	(0.17, 0.80)	0.010^*^
Age (years) *(ref. 16–25 years)*	26–30	1.08	(0.65, 1.78)	0.771
	31–35	1.03	(0.61, 1.75)	0.899
	36–40	0.71	(0.39, 1.28)	0.250
	41–50	0.66	(0.37, 1.19)	0.170
	50+	0.37	(0.17, 0.79)	0.010^*^
Ethnicity *(ref. Chinese)*	Malay	0.54	(0.28, 1.03)	0.049^*^
	Indian	0.71	(0.32, 1.60)	0.410
	Others	1.10	(0.56, 2.18)	0.780
Nationality *(ref. Singaporean)*	Singapore PR	0.88	(0.59, 1.31)	0.520
	Malaysian	0.86	(0.53, 1.39)	0.540
	Filipino	0.79	(0.34, 1.84)	0.600
	Others	0.78	(0.39, 1.55)	0.480
Education completed (*ref. University*)	A levels/polytechnic diploma	0.77	(0.53, 1.11)	0.160
	PSLE O levels/ITE diploma	0.78	(0.50, 1.22)	0.270
Employment *(ref. employed full/part-time)*	Student	1.15	(0.69, 1.93)	0.590
	Unemployed/retired	0.86	(0.47, 1.56)	0.620
Marital status *(ref. married/partner)*	Single/separated/divorced/widowed	0.80	(0.57, 1.12)	0.190
Known HIV status *(ref. No)*	Yes	1.24	(0.91, 1.69)	0.170
Substance used during sex (*ref. none)*	Alcohol and drugs	2.21	(1.62, 3.01)	<0.001^*^
	Alcohol only	1.77	(1.12, 2.82)	0.020^*^

Logistic regression model factoring in latent class was adjusted for age, ethnicity, education, nationality, employment, marital status, known HIV status, and substance use during sex (37 observations were dropped due to missing data). aOR, adjusted odds ratio; PR, permanent resident; PSLE, primary school leaving examination; O-level, ordinary level; ITE, Institute of Technical Education. The values with an “*” indicate variables for which significance was *p* < 0.05.

MSM using alcohol and drugs during sex had significantly higher odds of reporting unprotected sex (aOR = 2.3; 95% CI: 1.7–3.1) than non-users, as did those engaging in group sex (aOR = 2.2; 95% CI: 1.7–2.9), but there were no between-class differences (Table 5). Education was significantly associated with unprotected sex, and MSM with secondary level education had higher odds of unprotected sex (aOR = 2.0; 95% CI: 1.3–3.0) compared to university graduates. Being aged over 50 years and being single were negatively associated with unprotected sex.

**Table 5:** Model 3 outcome: unprotected anal sex in last 6 months.

		**aOR**	**95% CI**	***p*-value**
Latent class (*ref. Platform inactive)*	Sauna-centric	0.90	(0.63, 1.29)	0.580
	App centric	0.83	(0.57, 1.20)	0.320
	Multiple platforms	0.77	(0.47, 1.26)	0.299
	Do not “hook up”	1.07	(0.61, 1.90)	0.810
Age (years) *(ref. 16–25 years)*	26–30	0.96	(0.60, 1.54)	0.860
	31–35	0.91	(0.55, 1.49)	0.700
	36–40	0.77	(0.44, 1.35)	0.360
	41–50	0.80	(0.46, 1.38)	0.420
	50+	0.27	(0.13, 0.56)	<0.001^*^
Ethnicity *(ref. Chinese)*	Malay	1.47	(0.83, 2.59)	0.190
	Indian	1.01	(0.51, 2.01)	0.970
	Others	0.81	(0.43, 1.54)	0.520
Nationality *(ref. Singaporean)*	Singapore PR	1.11	(0.76, 1.61)	0.590
	Malaysian	0.76	(0.46, 1.24)	0.270
	Filipino	1.55	(0.62, 3.89)	0.350
	Others	0.97	(0.50, 1.89)	0.940
Education completed (*ref. University*)	A levels/polytechnic diploma	0.92	(0.65, 1.29)	0.641
	PSLE O levels/ITE diploma	1.94	(1.27, 2.97)	0.002^*^
Employment *(ref. employed full/part-time)*	Student	1.31	(0.79, 2.15)	0.300
	Unemployed/retired	1.35	(0.76, 2.42)	0.310
Marital status *(ref. married/partner)*	Single/separated/divorced/widowed	0.52	(0.37, 0.73)	<0.001^*^
Known HIV status *(ref. No)*	Yes	1.22	(0.91, 1.64)	0.180
Substance used during sex (*ref. none)*	Alcohol & drugs	2.29	(1.67, 3.14)	<0.001^*^
	Alcohol only	1.00	(0.63, 1.61)	0.998
Engaged in group sex *(ref. No)*	Yes	2.16	(1.63, 2.86)	<0.001^*^

Logistic regression model factoring in latent class was adjusted for age, ethnicity, education, nationality, employment, marital status, recent HIV testing, and substance use during sex (37 observations were dropped due to missing data). aOR, adjusted odds ratio; PR, permanent resident; PSLE, primary school leaving examination; O-level, ordinary level; ITE, Institute of Technical Education. The values with an “*” indicate variables for which significance was *p* < 0.05.

**Figure 2:**
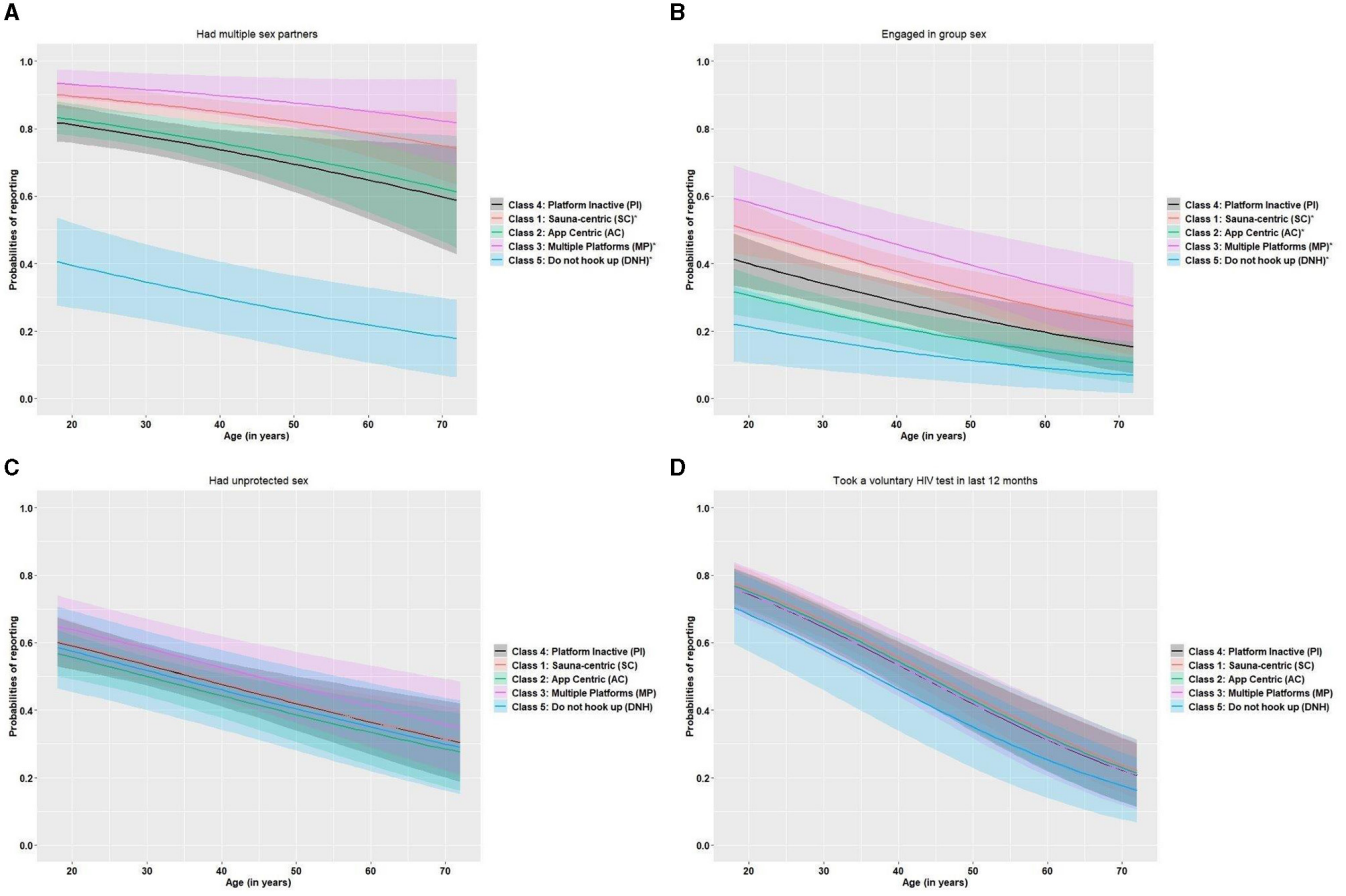
**(A–D)** Predicted probabilities for each behavioural outcome: **(A)** multiple sex partners, **(B)** engaging in group sex, **(C)** unprotected anal sex, and **(D)** recent HIV testing. The predicted probabilities for each outcome are plotted by latent class and participant age with 95% confidence intervals. In each plot, classes with “*” have a significant association with the outcome, compared to the platform inactive (PI) class. **(A)** For example, shows the probabilities of reporting multiple sex partners by class membership and by age (= relative position), where the sauna-centric (SC) and multiple-platform (MP) class membership are significantly associated with reporting multiple sex partners (compared to the platform inactive class).

Factors positively associated with recent HIV testing included engaging in group sex (aOR = 1.6; 95% CI: 1.2–2.1), alcohol and drug use during sex (aOR = 1.5; 95% CI: 1.1–2.1), being Singaporean or permanent resident (aOR = 2.0; 95% CI: 1.3–3.0), and disclosing one's sexual orientation (Table 6). The odds of recent testing for HIV were significantly higher in MSM who had disclosed their sexual orientation to two (aOR = 1.7; 95% CI: 1.08–2.64), three (aOR = 2.2; 95% CI: 1.2–4.1), and four groups (aOR = 2.6; 95% CI: 1.6–4.4) compared to none. Respondents aged 41–50 years and those over 50 years had significantly lower odds of recent HIV testing (aOR = 0.5; 95% CI: 0.3–0.8 and aOR = 0.2; 95% CI: 0.1–0.4, respectively). The predicted probabilities for each of the four behavioural outcomes (multiple sex partners, group sex, unprotected sex, and recent HIV testing) are plotted by age and latent class in Figures 2A–D.

**Table 6:** Model 4 outcome: HIV test in last 12 months.

		**aOR**	**95% CI**	***p*-value**
Latent class (*ref. Platform inactive)*	Sauna-centric	1.13	(0.77, 1.64)	0.540
	App-centric	0.97	(0.65, 1.44)	0.870
	Multiple platforms	0.76	(0.46, 1.26)	0.290
	Do not “hook up”	0.96	(0.51, 1.80)	0.890
Age (years) *(ref. 16–25 years)*	26–30	1.10	(0.67, 1.81)	0.710
	31–35	0.95	(0.56, 1.59)	0.840
	36–40	0.72	(0.40, 1.29)	0.270
	41–50	0.49	(0.28, 0.86)	0.011^*^
	50+	0.19	(0.09, 0.40)	<0.001
Ethnicity *(ref. Chinese)*	Malay	0.73	(0.41, 1.29)	0.280
	Indian	0.80	(0.39, 1.61)	0.530
	Others	1.12	(0.54, 2.29)	0.770
Nationality *(ref. Singaporean)*	Singapore PR	2.00	(1.33, 3.01)	0.002^*^
	Malaysian	1.06	(0.66, 1.71)	0.790
	Filipino	1.69	(0.66, 4.32)	0.270
	Others	1.30	(0.62, 2.70)	0.480
Education completed (*ref. University*)	A levels/polytechnic diploma	1.28	(0.90, 1.81)	0.169
	PSLE O levels/ITE diploma	0.69	(0.46, 1.04)	0.077
Employment *(ref. employed full/part-time)*	Student	0.66	(0.40, 1.10)	0.110
	Unemployed/retired	0.84	(0.45, 1.57)	0.580
Marital status *(ref. married/partner)*	Single/separated/divorced/widowed	1.12	(0.80, 1.57)	0.503
Disclosure of sexual orientation *(ref. Not disclosed to any)*	Disclosed to one group	1.23	(0.84, 1.80)	0.290
	Disclosed to two groups	1.69	(1.08, 2.64)	0.020^*^
	Disclosed to three groups	2.19	(1.17, 4.10)	0.010^*^
	Disclosed to four groups	2.61	(1.55, 4.41)	<0.001^*^
Substance(s) used during sex (*ref. none)*	Alcohol and drugs	1.49	(1.07, 2.08)	0.020^*^
	Alcohol only	1.44	(0.84, 2.46)	0.190
Engaged in group sex *(ref. No)*	Yes	1.56	(1.17, 2.10)	0.003^*^

Logistic regression model factoring in latent class was adjusted for age, ethnicity, education, nationality, employment, marital status, disclosure, group sex, and substance use during sex (37 observations were dropped due to missing data). aOR, adjusted odds ratio; PR, permanent resident; PSLE, primary school leaving examination; O-level, ordinary level; ITE, Institute of Technical Education. The values with an “*” indicate variables for which significance was *p* < 0.05.

## Discussion

We empirically characterised complex platform use patterns among MSM in Singapore and identified five distinct classes of platform use. Between-class differences in HIV-related risk behaviours and demographic factors suggest significant sub-group heterogeneity, which could better inform programmatic action and surveillance.

The *sauna-centric* and *multiple-platform* classes had similar probabilities of using saunas as a meeting platform. However, men in the SC class were older, exclusively used saunas and no other platform, and were more likely to *not* have disclosed their sexual orientation. By contrast, men in the MP class had high probabilities of meeting other men via internet or smartphone applications, in saunas, and on *most* other meeting platforms. Men in this class were more likely to have disclosed their sexual orientation, suggesting a greater degree of connectedness.

The SC (largest) and MP classes of respondents were significantly more likely to have *multiple sex partners*, used alcohol and drugs during sex, and to be single, separated, or divorced. SC and MP class membership, alcohol-only use during sex, and alcohol and drug use during sex were also independently associated with *group sex in the last 6 months*. Older age and being of Malay ethnicity were protective factors for group sex.

That the MP class was associated with multiple sex partners, group sex, and substance use is consistent with other studies of men seeking partners via the internet who reported more partners, group sex, condom-less sex, and a higher prevalence of chlamydia and gonorrhoea (10, 34). Fixed physical venues such as saunas, on the other hand, may be preferred for their anonymity by men with smaller networks who have not or prefer not to disclose their sexual orientation.

Alcohol *and* drug use during sex was significantly associated with MP class membership and was generally high in the sample (26%). The prevalence of sexualised drug use ranged from 3 to 13% in surveys across European cities and from 17 to 27% in men attending outpatient sexual health clinics (35–37). Sexualised drug use is a risk factor for not using condoms and acquiring bacterial sexually transmitted infections in HIV-negative MSM, and it has been associated with younger age, being single, and reporting other high-risk sex, including sex in exchange for goods/money (36–38).

Despite half the sample reporting *unprotected anal sex* in the last 6 months, latent class was not associated with not using a condom; however, lower education, group sex, and alcohol *and* drugs during sex remained significant risk factors. Older age and being single were protective of condom-less sex.

Predictors of *recent HIV testing* included group sex, alcohol and substance use during sex, being Singaporean or a permanent resident, and disclosure of sexual orientation to two or more groups, which suggests a heightened awareness of risk in those more connected. Higher HIV testing rates and knowledge of HIV status were reported among internet-using MSM in Europe (10, 11). No specific platform use pattern was associated with *recent HIV testing* but being above the age of 40 years and not having disclosed one's sexual orientation were negatively associated with recent HIV testing. A previous study reported non-disclosure to be negatively associated with HIV testing and underlines how the need to conceal stigmatised behaviours (i.e., sexual orientation) drives certain groups out of sight and out of reach of programmes and leads to lower awareness and possibly perception of risk (39). The repeal in 2022 of Section 377A of the Singapore Penal Code, criminalising sex between men, should make it easier over time for programmes to reach harder-to-reach sub-groups with more tailored HIV prevention education and testing, including on the risks of sexualized drug use (35, 40).

While certain meeting venues have been associated with high-risk behaviours among MSM, our findings suggest that considering the *mix* of platforms may provide a better reflection of sexual networks and HIV risk across platforms. Essentially, it can offer programmes more granular data to inform their differentiated service delivery (7, 41, 42). In the SC class, for example, prevention interventions need to account for an older demographic, non-disclosure, and possible language differences, which presents a unique opportunity to promote self-testing for HIV as well as pre-exposure prophylaxis and condom use. The availability of HIV self-test kits could be piloted in venues like saunas to expand testing. Prevention and lowering the risks of sexualised drug use and group sex needs to cut across platforms with messaging on condom use and PrEP intensified to mitigate infection risk, in concert with other substance use interventions (43). Barriers to the use and knowledge of PrEP would need to be explored among both SC and MP classes.

Finally, in settings like Singapore, low HIV prevalence can mask other STIs resulting from changes in sexual risk practices. Strengthening behavioural as well as STI surveillance in combination with relevant research approaches can provide programmes with better tools to monitor trends and target efforts.

Several strengths and limitations are acknowledged. Our sample may not be representative of all MSM as it relied on purposive sampling to recruit relatively hidden individuals. Nevertheless, by recruiting a large sample using several known meeting venues (saunas, bars/clubs, and online platforms), our reach in different MSM networks was increased. Recruitment for the survey was interrupted briefly between May and June 2020 due to restrictions implemented during a surge in COVID-19 cases locally; this finding may have led to some under-reporting of risk behaviours in the following period. Despite ensuring the survey was conducted in confidence by known and trusted community interviewers and in settings familiar to participants to minimise under-reporting of risk behaviours, bias towards socially desirable answers remains possible.

The strengths of our analysis are its relatively large sample size, its reach into the MSM community, and completeness of the data, which increase confidence in our results. Nevertheless, it is possible that platform use patterns measured in the short term may not be consistent longitudinally, be a good predictor of risk over time, or reflect cumulative exposure to certain risk factors. Repeat or longitudinal studies may contribute to establishing whether platform use evolves over time and how risk might change over the lifetime.

## Conclusion

Our analysis suggests that the *mix* of platforms used to meet sex partners provides an indicator of heterogeneity in HIV risk and sexual networks, especially in low HIV prevalence settings. This analytical approach combined with field knowledge strengthens evidence to target services in a differentiated manner. HIV self-test kits especially could be piloted in saunas used by older MSM who have not disclosed their orientation and whose first language is not English. Prevention to lower the risks of sexualised drug use and group sex can be tailored to different sub-group characteristics and messaging on condom use and PrEP intensified to mitigate infection risk, in concert with other substance use interventions.

## Data availability statement

The datasets generated and analysed during the current study are not publicly available due to them containing information that could compromise participant privacy. However, anonymised data may be made available by request to MT, National Public Health Epidemiology Unit, National Centre for Infectious Diseases. Requests to access these datasets should be directed to matthias_hs_toh@ncid.sg.

## Ethics statement

Studies involving humans are approved by the Domain Specific Review Board, National Health Care Group, Singapore. The study was conducted in accordance with the local legislation and institutional requirements. Written informed consent for participation was obtained from participants in accordance with the national legislation and institutional requirements.

## Author contributions

IT-E: Conceptualisation, Data curation, Formal analysis, Methodology, Writing – original draft, Writing – review & editing. YH: Data curation, Formal analysis, Methodology, Validation, Visualisation, Writing – original draft, Writing – review & editing. MI-CC: Conceptualisation, Writing – review & editing. CW: Validation, Writing – review & editing. CK: Data curation, Supervision, Validation, Writing – review & editing. MT: Resources, Validation, Writing – review & editing.
