# Supplementary material for: Can platform use patterns be an indicator of HIV-related risk and sub-group heterogeneity among men who have sex with men in Singapore: a latent class analysis
Source: Front Public Health. 2024 Apr 26;12:1330282. doi: 10.3389/fpubh.2024.1330282 (PMC11082277; doi:10.3389/fpubh.2024.1330282)
Supplement: Supplementary file 1 [file Data_Sheet_1.docx]

Supplementary Material

Title: Can platform use patterns be an indicator of HIV-related risk and sub-group heterogeneity among men who have sex with men in Singapore: a Latent Class Analysis

Isabel TAVITIAN-EXLEY^1†*^, Ying HAO^1†^, Mark I-C CHEN^1^, Chen Seong WONG^2^, Chronos KWOK^3^, Matthias Paul Han Sim TOH ^1,4^

^1^ National Public Health and Epidemiology Unit, National Centre for Infectious Diseases, Singapore.

^2^ National HIV Programme, National Centre for Infectious Diseases, Singapore

^3^ Action for AIDS Singapore, Singapore

^4^ Saw Swee Hock School of Public Health, National University of Singapore, Singapore

†These authors have contributed equally to the work

**Supplementary Table 1: Additional details on survey recruitment methods and results**

**Survey and recruitment methods**

“The ninth round of outreach HIV testing project in MSM venues was held in 2020. The first project was conducted in November 2007 and has since been repeated almost every year up till 2016. Each project lasted for about two and a half months and was conducted by Action for AIDS (AfA), Singapore. The project did not run from 2017 to 2019 but was put up for funding in early 2020. The funding approval was delayed due to the COVID-19 pandemic and was finally approved in the last quarter of 2020 when the project commenced once again”.

***Target population, recruitment process, recruitment time and data collection***

“The target sample in 2020 was 1,300 men who have sex with men (MSM) based on a minimum sample size of 1169, calculated using the highest sero-positivity of 3.14% recorded in 2013 with a 1% margin of error and 95% level of confidence, and allowing for spoilt test kits and specimens…. The recruitment targets by “venue” was set at 500 for bars/clubs, 500 for saunas and 300 on-line.“

“All men who have sex with men identifying as gay, bisexual or of queer sexual orientation and aged 18 years and above were eligible to take part in the study and given a participant information sheet. Eligible participants were given the opportunity to ask questions and provide consent for their participation in the study electronically by clicking on a button to indicate their agreement to their participation in the study. Participants were asked to respond to an iPad-administered survey in private, while the AfA volunteer stood by to assist in the event any clarification was required. Participants were offered HIV and Syphilis testing free of charge. The survey form included standard demographic information such as age, educational attainment, ethnicity, nationality and on sexual behaviours and practices as well as access to, and use of, services.”

“All MSM at saunas, clubs and bars were invited to participate in this project during which the Mobile Testing Service (MTS) was deployed for sessions at the bars/clubs and saunas. Recruitment online was done via mobile applications networking apps such as Grindr and Jack’d where a banner or pop-up ad invited interested participants to the survey. For those who registered and completed the survey, a unique code was supplied allowing them to go to one of the Anonymous Testing Sites and receive free testing and counselling for HIV and Syphillis.”

**Supplementary Table 2: Questionnaire item with nine original response options**

**Recruitment results**

*Recruitment during sauna outreach sessions* – “there was no difficulty getting all four existing sauna proprietors to participate in this ninth round given the MSM community’s familiarity with the project and awareness of HIV and AIDS. Some sauna owners helped to promote testing dates to their patrons. Testing was conducted at the lounge area of the saunas. A total of 21 sauna sessions were conducted with each session lasting around 2 to 2.5 hours. On average, each sauna session received about 20 to 25 participants.

*Recruitment during for club outreach sessions* - the project team was unable to physically enter bars and clubs to recruit participants due to COVID-19 safe distancing measures. Instead, publicity was done on AfA’s Gayhealth.sg, Facebook and Instagram accounts to inform the MSM community that a free HIV/Syphilis testing service was provided on the publicised dates. A total of 9 club outreach sessions were conducted. Each session lasted around 2.5 hours and were quite well received with an average of 50 participants recruited into the survey per session.

*Recruitment via the dating app* - a paid advertising campaign was run on Jack’d - a popular MSM dating app with 6,300 active monthly users. Through the campaign, users were prompted to fill in the survey and were subsequently emailed a free testing coupon which could be redeemed at the ATS. Over 600 Jack’d users completed the survey and were sent the free testing coupon, of these 184 participants turned up at ATS to get tested.

*Sample collection at Clubs* - This was the first time the project used the HIV Gen 4 blood test kit in place of the OraQuick test kit. Due to the requirement for blood taking, AfA’s mobile testing van was used for the club outreach sessions. The sample collection for both HIV and Syphilis tests were conducted inside the mobile testing van. The van was parked at the same location for all the club outreach sessions - along Neil Road within walking distance from the clubs.

*Testing at saunas* - as in previous years, testing at saunas were held at the lounge area. However, due to the sample collection requirements, the project team worked with sauna owners to ensure that the testing area had sufficient space and light for the pricker to conduct the tests. This was to ensure that the process could be conducted in a safe manner and for the test results to be accurately read.

Participants from clubs and saunas were each given a unique serial numbered ID receipt (Annex A) with a telephone number to call for test results and additional counselling if required. The unique serial numbers were printed on stickers, and these were stuck on both the test kits as well as receipts that were handed out. Participants were given the option to call back after 30 minutes or to collect their result personally by showing their receipt to the AfA staff on site. The tests were done anonymously to minimize the fear of stigmatization and loss of confidentiality, and to maximize uptake. Lastly, to ensure that confidentiality was not breached when a participant tested positive, all results were delivered by a trained member of AfA staff.”

*Source: Gay men’s health survey report (unpublished)*

| # | Question | **Response categories** |
| --- | --- | --- |
| 13. | Where/how do you typically meet male sex partner(s)? | **Please select all that apply:**  Not applicable (Do not hook up) _(1)_  Bars and clubs _(2)_  Saunas _(3)_  Public spaces (e.g. parks, gyms, restrooms) _(4)_  Private or home parties _(5)_  Through internet sites and chatrooms_(6)_ **(If select, go to Q13)**  Through Smartphone Apps _(7)_ **(If select, go to Q13)**  Through friends _(8)_  Paid someone to have sex (e.g. escorts, masseurs) _(9)_ |

**Supplementary Table 3: Overall sample characteristics**

| **Variables** (n, col %) | | **All MSM** (N=1141~~, %~~) | | |
| --- | --- | --- | --- | --- |
| **Recruitment venues** | Bars/Clubs (3) | 426 | | 37% |
|  | Sauna (4) | 531 | | 47% |
|  | Smartphone App (1, Jack’d) | 184 | | 16% |
| **Demographics** |  |  | |  |
| **Age** *(mean (SD) min-max)* | | 33 (10.1) | | (18-72) |
| **Age** (years) | 16-25 | 234 | | 21% |
|  | 26-30 | 290 | | 25% |
|  | 31-35 | 238 | | 21% |
|  | 36-40 | 141 | | 12% |
|  | 41-50 | 151 | | 13% |
|  | 50+ years old | 87 | | 8% |
| **Ethnicity** | Chinese | 934 | | 82% |
|  | Malay | 72 | | 6% |
|  | Indian | 43 | | 4% |
|  | Other | 92 | | 8% |
| **Education** | PSLE/O-Levels/ITE | 170 | | 15% |
|  | A Levels/Polytechnic, Diploma | 261 | | 24% |
|  | University and above | 673 | | 61% |
|  | Prefer not to answer | 37 | | 3% |
| **Nationality** | Singaporean | 771 | | 68% |
|  | Singapore PR | 160 | | 14% |
|  | Malaysian | 106 | | 9% |
|  | Filipino | 42 | | 4% |
|  | Others | 62 | | 5% |
| **Marital status** | Single/Separated/Divorced | 919 | | 81% |
|  | Partnered/Married | 222 | | 19% |
| **Employment status** | Full time/part time employment | 898 | | 79% |
|  | Student | 136 | | 12% |
|  | Unemployed/retired | 68 | | 6% |
|  | Prefer not to answer | 39 | | 3% |
| **Disclosure** | No disclosure | 219 | | 19% |
|  | One group | 442 | | 39% |
|  | ≥ Two groups | 480 | | 42% |
| **Risk behaviours** |  |  | |  |
| ≥ 2 sex partners | Yes | 904 | | 79% |
|  | No | 237 | | 21% |
| Unprotected anal sex | Yes | 581 | | 51% |
|  | No | 560 | | 49% |
| Engaged in group sex | Yes | 392 | | 34% |
|  | No | 749 | | 66% |
| Substance use during sex | Alcohol & drugs | 298 | | 26% |
|  | Alcohol only | 93 | | 8% |
|  | None | 750 | | 66% |
| **Testing and treatment** | Test for HIV in past 12 months | 692 | | 61% |
|  | Test for syphilis in last 6 months | 339 | | 30% |
|  | STI diagnosis in past 6 months | 105 | | 9% |
| **Pre-Exposure Prophylaxis** | Has heard of PrEP | 1,022 | | 90% |
|  | Has taken PrEP | 247 | | 22% |
| **Serological markers** | HIV test positive | 8 | 0.7% | |
|  | Syphilis reactive | 12 | 1.1% | |
|  | HIV +ve or syphilis reactive | 19 | 1.7% | |

Note: HIV Human Immune-Deficiency Virus. STI=Sexually transmitted infection. PrEP=Pre-exposure Prophylaxis. PSLE= Primary School Leaving Examination (equivalent to primary school education). ITE=Institute of Technical Education (equivalent to secondary school education). Singapore PR=Singapore Permanent Resident

Supplementary Table 4: Fit statistics and indices for a latent class analysis of nine indicators in men who have sex with men

| **Fit statistic** | **C2 Model** | **C3 Model** | **C4 Model** | **C5 Model** | **C6 Model** |
| --- | --- | --- | --- | --- | --- |
| **AIC** | 8756.9 | 8559 | 8313.2 | 8246.9 | 8214.4 |
| **BIC** | 8852.7 | 8705.1 | 8509.7 | 8493.8 | 8511.7 |
| **Pearson’s X^2^** | <0.001 | <0.001 | 0.929 | 1 | 1 |
| **LR test X^2^** | <0.001 | <0.001 | <0.001 | <0.001 | <0.001 |
| **LL** | -4359.5 | -4250.5 | -4117.6 | -4074.4 | -4048.2 |
| **Entropy** | 0.8 | 0.9 | 1.0 | 1.0 | 1.0 |

Notes on model fit statistics for Latent class models with nine binary variables. AIC= Akaike information criteria. BIC= and Bayesian information criteria. Test p-values shown for Pearson’s X2, Likelihood ratio (LR) which compares current n class to n-1 class model. Model set and replicated at 400 random starts.

Interpretation of latent class analysis model fit statistics:

A non-significant value for the LRT suggests that the model with one fewer class better explained the data and a smaller AIC and BIC indicated a better model fit (1-3). Entropy provides a measure of the degree to which latent classes are distinct from each other, by estimating individual conditional probabilities of class membership to assess the precision of class assignment and thus the usefulness and value of the resulting classes. Preference was given to entropy statistic values closest to +1 indicating greater entropy (1, 3). Individuals within a given class or sub-type were considered homogenous when they had similar item responses and when class-specific response probabilities for binary indicators were above 0.70 or below 0.30 (1). Models were estimated using maximum likelihood with a minimum of 400 random starts to ensure that global maxima solutions were reached (4). Conditional probabilities are the posterior probabilities of endorsing a meeting platform variable for an individual classified in their most likely class in the five-class model.

**References**

1. Roesch SC, Villodas M, Villodas F. Latent class/profile analysis in maltreatment research: A commentary on Nooner et al., Pears et al., and looking beyond. Child Abuse Negl. 2010;34(3):155-60.

2. Lanza ST, Bray BC, Collins LM. An Introduction to Latent class and Latent transition analysis. 2013. In: Handbook of Psychology, Vol 2: Research Methods in Psychology [Internet]. Hoboken, NJ, US: Wiley. 2nd. [691-715].

3. Flaherty BP, Kiff CJ. Latent class and latent profile models. In: Cooper H, Camic PM, Long DL, Panter AT, Rindskopf D, Sher KJ, editors. APA handbook of research methods in psychology, Vol 3: Data analysis and research publication. Washington, DC, US: American Psychological Association; 2012. p. 391-404.

4. Muthén LK, Muthén BO. Mplus User’s Guide. . Los Angeles, CA: Muthén & Muthén; 1998-2012.
